# 4-Chloro-*N*-(3,4-dimethyl­phen­yl)benzamide

**DOI:** 10.1107/S1600536810014972

**Published:** 2010-04-28

**Authors:** B. Thimme Gowda, Sabine Foro, Vinola Z. Rodrigues, Hartmut Fuess

**Affiliations:** a Department of Chemistry, Mangalore University, Mangalagangotri 574 199, Mangalore, India; b Institute of Materials Science, Darmstadt University of Technology, Petersenstrasse 23, D-64287 Darmstadt, Germany

## Abstract

In the title compound, C_15_H_14_ClNO, the N—H bond is *trans* to the C=O bond. The dihedral angle between the two aromatic rings is 5.5 (2)°. In the crystal, inter­molecular N—H⋯O hydrogen bonds link the mol­ecules into chains running along the *a* axis.

## Related literature

For the preparation of the title compound, see: Gowda *et al.* (2003 ▶). For related structures, see: Bowes *et al.* (2003 ▶); Gowda *et al.* (2008**a* ▶,b* ▶, 2009 ▶).
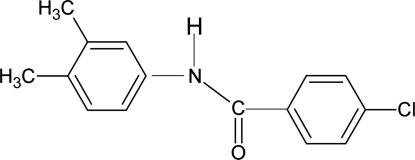


## Experimental

### 

#### Crystal data

C_15_H_14_ClNO
*M* *_r_* = 259.72
Orthorhombic, 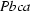

*a* = 9.550 (1) Å
*b* = 10.104 (2) Å
*c* = 28.133 (4) Å
*V* = 2714.6 (7) Å^3^
*Z* = 8
Mo *K*α radiation
μ = 0.27 mm^−1^
*T* = 299 K
0.38 × 0.20 × 0.06 mm

#### Data collection

Oxford Diffraction Xcalibur diffractometer with a Sapphire CCD detector
Absorption correction: multi-scan (*CrysAlis RED*; Oxford Diffraction, 2009 ▶) *T*_min_ = 0.905, *T*_max_ = 0.984
9354 measured reflections
2469 independent reflections
1405 reflections with *I* > 2σ(*I*)
*R*_int_ = 0.051

#### Refinement

*R*[*F* ^2^ > 2σ(*F* ^2^)] = 0.086
*wR*(*F* ^2^) = 0.193
*S* = 1.16
2469 reflections
166 parameters
1 restraint
H atoms treated by a mixture of independent and constrained refinement
Δρ_max_ = 0.23 e Å^−3^
Δρ_min_ = −0.23 e Å^−3^

### 

Data collection: *CrysAlis CCD* (Oxford Diffraction, 2009 ▶); cell refinement: *CrysAlis RED* (Oxford Diffraction, 2009 ▶); data reduction: *CrysAlis RED*; program(s) used to solve structure: *SHELXS97* (Sheldrick, 2008 ▶); program(s) used to refine structure: *SHELXL97* (Sheldrick, 2008 ▶); molecular graphics: *PLATON* (Spek, 2009 ▶); software used to prepare material for publication: *SHELXL97*.

## Supplementary Material

Crystal structure: contains datablocks I, global. DOI: 10.1107/S1600536810014972/bt5253sup1.cif

Structure factors: contains datablocks I. DOI: 10.1107/S1600536810014972/bt5253Isup2.hkl

Additional supplementary materials:  crystallographic information; 3D view; checkCIF report

## Figures and Tables

**Table 1:** Hydrogen-bond geometry (Å, °)

*D*—H⋯*A*	*D*—H	H⋯*A*	*D*⋯*A*	*D*—H⋯*A*
N1—H1*N*⋯O1^i^	0.86 (1)	2.04 (2)	2.862 (5)	160 (4)

Symmetry code: (i) 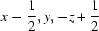.

**Fig. 1.**
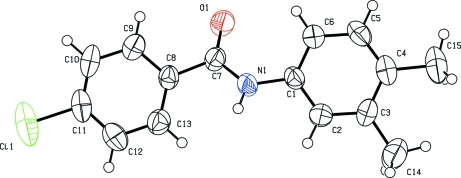
Molecular structure of the title compound, showing the atom labelling scheme. The displacement ellipsoids are drawn at the 50% probability level. The H atoms are represented as small spheres of arbitrary radii.

**Fig. 2.**
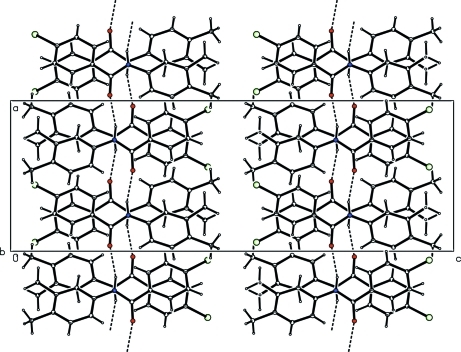
Molecular packing of the title compound with hydrogen bonding shown as dashed lines.

## supplementary crystallographic information

### Comment

In the present work, as part of a study of the substituent effects on the crystal structures of benzanilides (Gowda *et al.*, 2008*a,b*, 2009), the structure of *N*-(3,4-dimethylphenyl)-4-chlorobenzamide (I) has been determined. In the structure, the conformations of the N—H and C=O bonds are *anti* to each other(Fig. 1), similar to those observed in *N*-(3,4-dimethylphenyl)-4-methylbenzamide(II)(Gowda *et al.*, 2009), *N*-(3,4-dimethylphenyl)benzamide(III)(Gowda *et al.*, 2008*a*), *N*-(2,6-dimethylphenyl)4-chlorobenzamide (IV)(Gowda *et al.*, 2008*b*) and the parent benzanilide (Bowes *et al.*, 2003). Further, the conformation of the N—H bond is *syn* to the *meta*- methyl-substituent in the anilino ring.

The dihedral angle between the two benzene rings is 5.5 (2)°, compared to the values of 52.6 (1)° and 10.5 (1)° in the two molecules of (II), and 39.9 (2)°, 51.0 (1)° and 87.2 (3)° in the molecules 1, 2 and 3 of (IV), respectively.

The packing diagram of molecules in (I) showing the intermolecular N–H···O hydrogen bonds (Table 1) involved in the formation of molecular chains running along the *a*-axis is shown in Fig. 2.

### Experimental

The title compound was prepared according to the literature method (Gowda *et al.*, 2003). The purity of the compound was checked by determining its melting point. It was characterized by recording its infrared and NMR spectra.

Needle like colourless single crystals of the title compound used in X-ray diffraction studies were obtained from a slow evaporation of its ethanolic solution at room temperature.

### Refinement

The H atom of the NH group was located in a difference map. It was refined with the distance  restrained to N—H = 0.86 (1) %A. The other H atoms were positioned with idealized geometry using a riding model with C—H = 0.93–0.96 Å. All H atoms were refined with isotropic displacement parameters set to 1.2 times of the *U*_eq_ of the parent atom.

### Crystal data

**Table:**

C_15_H_14_ClNO	*F*(000) = 1088
*M**_r_* = 259.72	*D*_x_ = 1.271 Mg m^−^^3^
Orthorhombic, *P**b**c**a*	Mo *K*α radiation, λ = 0.71073 Å
Hall symbol: -P 2ac 2ab	Cell parameters from 2409 reflections
*a* = 9.550 (1) Å	θ = 2.6–27.9°
*b* = 10.104 (2) Å	µ = 0.27 mm^−^^1^
*c* = 28.133 (4) Å	*T* = 299 K
*V* = 2714.6 (7) Å^3^	Needle, colourless
*Z* = 8	0.38 × 0.20 × 0.06 mm

### Data collection

**Table:**

Oxford Diffraction Xcalibur diffractometer with a Sapphire CCD detector	2469 independent reflections
Radiation source: fine-focus sealed tube	1405 reflections with *I* > 2σ(*I*)
graphite	*R*_int_ = 0.051
Rotation method data acquisition using ω and phi scans	θ_max_ = 25.3°, θ_min_ = 2.6°
Absorption correction: multi-scan (*CrysAlis RED*; Oxford Diffraction, 2009)	*h* = −11→10
*T*_min_ = 0.905, *T*_max_ = 0.984	*k* = −7→12
9354 measured reflections	*l* = −24→33

### Refinement

**Table:**

Refinement on *F*^2^	Primary atom site location: structure-invariant direct methods
Least-squares matrix: full	Secondary atom site location: difference Fourier map
*R*[*F*^2^ > 2σ(*F*^2^)] = 0.086	Hydrogen site location: inferred from neighbouring sites
*wR*(*F*^2^) = 0.193	H atoms treated by a mixture of independent and constrained refinement
*S* = 1.16	*w* = 1/[σ^2^(*F*_o_^2^) + (0.0485*P*)^2^ + 3.5049*P*] where *P* = (*F*_o_^2^ + 2*F*_c_^2^)/3
2469 reflections	(Δ/σ)_max_ = 0.003
166 parameters	Δρ_max_ = 0.23 e Å^−^^3^
1 restraint	Δρ_min_ = −0.23 e Å^−^^3^

### Special details

**Table:**

Experimental. CrysAlis RED (Oxford Diffraction, 2009) Empirical absorption correction using spherical harmonics, implemented in SCALE3 ABSPACK scaling algorithm.
Geometry. All esds (except the esd in the dihedral angle between two l.s. planes) are estimated using the full covariance matrix. The cell esds are taken into account individually in the estimation of esds in distances, angles and torsion angles; correlations between esds in cell parameters are only used when they are defined by crystal symmetry. An approximate (isotropic) treatment of cell esds is used for estimating esds involving l.s. planes.
Refinement. Refinement of *F*^2^ against ALL reflections. The weighted *R*-factor wR and goodness of fit *S* are based on *F*^2^, conventional *R*-factors *R* are based on *F*, with *F* set to zero for negative *F*^2^. The threshold expression of *F*^2^ > σ(*F*^2^) is used only for calculating *R*-factors(gt) etc. and is not relevant to the choice of reflections for refinement. *R*-factors based on *F*^2^ are statistically about twice as large as those based on *F*, and *R*- factors based on ALL data will be even larger.

### Fractional atomic coordinates and isotropic or equivalent isotropic displacement parameters (Å^2^)

**Table:**

	*x*	*y*	*z*	*U*_iso_*/*U*_eq_	
C1	0.3133 (4)	0.2816 (4)	0.31176 (13)	0.0477 (10)	
C2	0.2436 (5)	0.2315 (5)	0.35073 (13)	0.0576 (12)	
H2	0.1655	0.1782	0.3459	0.069*	
C3	0.2865 (5)	0.2583 (5)	0.39707 (13)	0.0581 (12)	
C4	0.4057 (5)	0.3357 (5)	0.40367 (15)	0.0602 (13)	
C5	0.4728 (5)	0.3856 (4)	0.36439 (16)	0.0611 (12)	
H5	0.5514	0.4386	0.3689	0.073*	
C6	0.4286 (5)	0.3605 (4)	0.31838 (14)	0.0551 (12)	
H6	0.4759	0.3964	0.2925	0.066*	
C7	0.3374 (4)	0.2232 (4)	0.22645 (13)	0.0500 (11)	
C8	0.2595 (5)	0.1668 (4)	0.18478 (12)	0.0457 (10)	
C9	0.3119 (5)	0.1932 (4)	0.13955 (13)	0.0564 (12)	
H9	0.3899	0.2475	0.1363	0.068*	
C10	0.2503 (6)	0.1405 (5)	0.09976 (14)	0.0663 (14)	
H10	0.2859	0.1590	0.0697	0.080*	
C11	0.1355 (6)	0.0602 (5)	0.10495 (15)	0.0655 (14)	
C12	0.0799 (5)	0.0327 (4)	0.14903 (17)	0.0664 (13)	
H12	0.0014	−0.0211	0.1520	0.080*	
C13	0.1436 (5)	0.0869 (4)	0.18880 (15)	0.0573 (12)	
H13	0.1072	0.0688	0.2188	0.069*	
C14	0.2071 (6)	0.2009 (6)	0.43859 (15)	0.0912 (18)	
H14A	0.2101	0.1061	0.4371	0.109*	
H14B	0.1114	0.2300	0.4374	0.109*	
H14C	0.2491	0.2302	0.4678	0.109*	
C15	0.4610 (6)	0.3627 (5)	0.45336 (16)	0.0898 (18)	
H15A	0.4821	0.2804	0.4688	0.108*	
H15B	0.3915	0.4095	0.4714	0.108*	
H15C	0.5445	0.4154	0.4513	0.108*	
N1	0.2614 (3)	0.2460 (4)	0.26579 (11)	0.0547 (10)	
H1N	0.1728 (14)	0.233 (4)	0.2629 (14)	0.066*	
O1	0.4632 (3)	0.2440 (4)	0.22403 (10)	0.0739 (10)	
Cl1	0.0598 (2)	−0.01106 (17)	0.05511 (5)	0.1200 (8)	

### Atomic displacement parameters (Å^2^)

**Table:**

	*U*^11^	*U*^22^	*U*^33^	*U*^12^	*U*^13^	*U*^23^
C1	0.038 (2)	0.061 (3)	0.044 (2)	−0.002 (2)	−0.0062 (18)	−0.007 (2)
C2	0.045 (3)	0.077 (3)	0.051 (2)	−0.007 (3)	0.001 (2)	−0.003 (2)
C3	0.066 (3)	0.070 (3)	0.038 (2)	0.007 (3)	0.002 (2)	−0.001 (2)
C4	0.072 (4)	0.059 (3)	0.050 (3)	0.007 (3)	−0.016 (2)	−0.010 (2)
C5	0.064 (3)	0.052 (3)	0.067 (3)	−0.012 (3)	−0.014 (3)	−0.009 (2)
C6	0.057 (3)	0.062 (3)	0.046 (2)	−0.009 (3)	−0.002 (2)	−0.002 (2)
C7	0.040 (3)	0.070 (3)	0.039 (2)	0.006 (2)	−0.0009 (19)	0.000 (2)
C8	0.042 (3)	0.056 (3)	0.039 (2)	0.008 (2)	−0.0043 (19)	−0.0027 (18)
C9	0.059 (3)	0.066 (3)	0.045 (2)	0.008 (3)	0.003 (2)	0.002 (2)
C10	0.089 (4)	0.076 (3)	0.034 (2)	0.018 (3)	−0.004 (2)	−0.005 (2)
C11	0.088 (4)	0.060 (3)	0.048 (3)	0.013 (3)	−0.020 (3)	−0.014 (2)
C12	0.072 (4)	0.054 (3)	0.074 (3)	−0.004 (3)	−0.013 (3)	−0.014 (2)
C13	0.063 (3)	0.060 (3)	0.050 (2)	0.002 (3)	0.005 (2)	−0.006 (2)
C14	0.096 (5)	0.125 (5)	0.052 (3)	−0.003 (4)	0.005 (3)	0.006 (3)
C15	0.128 (5)	0.084 (4)	0.058 (3)	0.001 (4)	−0.032 (3)	−0.014 (3)
N1	0.0341 (19)	0.087 (3)	0.0428 (18)	−0.007 (2)	−0.0019 (16)	−0.0121 (17)
O1	0.0312 (16)	0.138 (3)	0.0523 (18)	−0.003 (2)	0.0006 (13)	−0.0042 (18)
Cl1	0.1683 (18)	0.1139 (13)	0.0777 (10)	0.0066 (13)	−0.0518 (10)	−0.0319 (9)

### Geometric parameters (Å, °)

**Table:**

C1—C6	1.372 (5)	C9—C10	1.372 (6)
C1—C2	1.379 (5)	C9—H9	0.9300
C1—N1	1.431 (5)	C10—C11	1.372 (7)
C2—C3	1.393 (5)	C10—H10	0.9300
C2—H2	0.9300	C11—C12	1.378 (6)
C3—C4	1.394 (6)	C11—Cl1	1.734 (4)
C3—C14	1.508 (6)	C12—C13	1.386 (6)
C4—C5	1.374 (6)	C12—H12	0.9300
C4—C15	1.519 (6)	C13—H13	0.9300
C5—C6	1.385 (5)	C14—H14A	0.9600
C5—H5	0.9300	C14—H14B	0.9600
C6—H6	0.9300	C14—H14C	0.9600
C7—O1	1.221 (5)	C15—H15A	0.9600
C7—N1	1.344 (5)	C15—H15B	0.9600
C7—C8	1.500 (5)	C15—H15C	0.9600
C8—C13	1.376 (6)	N1—H1N	0.860 (10)
C8—C9	1.393 (5)		
			
C6—C1—C2	119.5 (4)	C9—C10—C11	119.0 (4)
C6—C1—N1	123.2 (4)	C9—C10—H10	120.5
C2—C1—N1	117.3 (4)	C11—C10—H10	120.5
C1—C2—C3	122.1 (4)	C10—C11—C12	121.6 (4)
C1—C2—H2	118.9	C10—C11—Cl1	119.5 (4)
C3—C2—H2	118.9	C12—C11—Cl1	118.9 (4)
C2—C3—C4	118.3 (4)	C11—C12—C13	118.5 (5)
C2—C3—C14	120.2 (4)	C11—C12—H12	120.7
C4—C3—C14	121.5 (4)	C13—C12—H12	120.7
C5—C4—C3	118.7 (4)	C8—C13—C12	121.3 (4)
C5—C4—C15	120.8 (5)	C8—C13—H13	119.4
C3—C4—C15	120.5 (5)	C12—C13—H13	119.4
C4—C5—C6	122.8 (4)	C3—C14—H14A	109.5
C4—C5—H5	118.6	C3—C14—H14B	109.5
C6—C5—H5	118.6	H14A—C14—H14B	109.5
C1—C6—C5	118.6 (4)	C3—C14—H14C	109.5
C1—C6—H6	120.7	H14A—C14—H14C	109.5
C5—C6—H6	120.7	H14B—C14—H14C	109.5
O1—C7—N1	123.2 (4)	C4—C15—H15A	109.5
O1—C7—C8	120.6 (4)	C4—C15—H15B	109.5
N1—C7—C8	116.2 (4)	H15A—C15—H15B	109.5
C13—C8—C9	118.5 (4)	C4—C15—H15C	109.5
C13—C8—C7	123.9 (4)	H15A—C15—H15C	109.5
C9—C8—C7	117.6 (4)	H15B—C15—H15C	109.5
C10—C9—C8	121.1 (5)	C7—N1—C1	126.9 (3)
C10—C9—H9	119.4	C7—N1—H1N	115 (3)
C8—C9—H9	119.4	C1—N1—H1N	118 (3)
			
C6—C1—C2—C3	−0.1 (7)	N1—C7—C8—C9	153.1 (4)
N1—C1—C2—C3	179.0 (4)	C13—C8—C9—C10	−0.3 (6)
C1—C2—C3—C4	−1.5 (7)	C7—C8—C9—C10	177.1 (4)
C1—C2—C3—C14	−179.8 (4)	C8—C9—C10—C11	−0.2 (7)
C2—C3—C4—C5	2.1 (6)	C9—C10—C11—C12	0.7 (7)
C14—C3—C4—C5	−179.6 (5)	C9—C10—C11—Cl1	−178.1 (3)
C2—C3—C4—C15	−177.2 (4)	C10—C11—C12—C13	−0.7 (7)
C14—C3—C4—C15	1.1 (7)	Cl1—C11—C12—C13	178.1 (3)
C3—C4—C5—C6	−1.1 (7)	C9—C8—C13—C12	0.3 (6)
C15—C4—C5—C6	178.1 (4)	C7—C8—C13—C12	−176.9 (4)
C2—C1—C6—C5	1.1 (6)	C11—C12—C13—C8	0.2 (7)
N1—C1—C6—C5	−178.0 (4)	O1—C7—N1—C1	−8.0 (8)
C4—C5—C6—C1	−0.5 (7)	C8—C7—N1—C1	170.8 (4)
O1—C7—C8—C13	149.2 (5)	C6—C1—N1—C7	34.7 (7)
N1—C7—C8—C13	−29.6 (6)	C2—C1—N1—C7	−144.4 (5)
O1—C7—C8—C9	−28.1 (6)		

### Hydrogen-bond geometry (Å, °)

**Table:**

*D*—H···*A*	*D*—H	H···*A*	*D*···*A*	*D*—H···*A*
N1—H1N···O1^i^	0.86 (1)	2.04 (2)	2.862 (5)	160 (4)

Symmetry codes: (i) *x*−1/2, *y*, −*z*+1/2.
